# Nicotine replacement therapy as a smoking cessation tool for adolescents: an update

**DOI:** 10.3389/fpsyt.2025.1525510

**Published:** 2025-02-25

**Authors:** Ioannis Beis, Anastasios Dimou, Serafeim-Chrysovalantis Kotoulas, Athanasia Pataka

**Affiliations:** ^1^ Respiratory Failure Unit, G. Papanikolaou Hospital, Aristotle University of Thessaloniki, Thessaloniki, Greece; ^2^ Urology Department, Venizeleio Hospital of Heralkion, Crete, Greece; ^3^ Intensive Care Unit (ICU), Ippokrateio General Hospital, Thessaloniki, Greece

**Keywords:** nicotine replacement therapy, adolescents, smoking cessation, e-cigarette, systematic review

## Abstract

**Background:**

Adolescent smoking is a significant public health concern, as early nicotine addiction leads to more severe addiction and reduced cessation success during adulthood. While nicotine replacement therapy (NRT) is an effective smoking cessation tool in adults, its efficacy in adolescents is less clear.

**Objective:**

This systematic review evaluates the effectiveness and safety of NRT for smoking cessation in adolescents.

**Methods:**

A comprehensive search of PubMed and Cochrane Library databases identified 12 studies (randomized controlled trials and observational) examining NRT in adolescents. Outcomes included smoking cessation rates, withdrawal symptom relief, smoking reduction, and adverse events.

**Results:**

NRT demonstrated limited success in long-term smoking cessation among adolescents, with low cessation rates that often declined post-treatment. However, NRT was effective in reducing smoking frequency and in managing withdrawal symptoms in some cases. The safety profile was generally favourable, with mild side effects such as skin irritation, headaches, and nausea.

**Conclusion:**

While NRT can reduce smoking and alleviate withdrawal symptoms, its effectiveness in sustaining long-term cessation in adolescents is limited. Adherence challenges and side effects suggest a need for complementary behavioural support and further research into tailored NRT strategies for this population.

## Introduction

Tobacco use remains a significant public health issue globally, with a worrying trend of initiation during adolescence. The Centers for Disease Control and Prevention (CDC) reports that nicotine addiction starts primarily in youth and young adults, with nearly 90% of adult smokers having initiated smoking by age 18 (1).

The smoking epidemic among teenagers is spreading rapidly at a period when social media use and online tobacco advertising thrive, contributing to lifetime tobacco use (2). Moreover, a rapid increase in novel tobacco product use is being observed among teenagers, often promoted by the tobacco industry itself (3). The latter has been a key driver in perpetuating nicotine addiction among teens, advertising e-cigarettes and other products as “less harmful” alternatives to traditional smoking (4).Factors that also can play a crucial role in developing nicotine addiction during adolescence include neurodevelopmental changes, susceptibility to risky behaviour, desire for experimentation, interaction and exposure to people with smoking habits, such as parents and peers, and finally, genetic predisposition (5–7).

It is a well-established fact that smoking at a young age is associated with greater addiction severity and decreased success in quitting later in life. It profoundly affects brain development, cognitive function, emotional regulation, and creates a potent nicotine dependence due to drug reward (8). Early intervention in adolescent smoking could significantly alter the trajectory of nicotine addiction, potentially reducing the prevalence of adult smoking and associated diseases. Interestingly, it has been found that adolescent smokers often express desire in quitting (9).

The standard smoking cessation interventions in adults involve first line pharmacotherapy, with either nicotine replacement therapy (NRT), bupropion or varenicline, and behavioural therapy (9, 10). In particular, the effectiveness of NRT therapy has long been evaluated and verified by studies in adult population and its products are widely used as a means for smoking cessation (11). NRT, which includes patches, gums, lozenges, inhalers, and nasal sprays, delivers lower levels of nicotine to help manage withdrawal symptoms and reduce the urge to smoke (12). Unlike tobacco products, NRT products do not contain tar or other harmful chemicals found in cigarettes and offer a promising tool, though its applicability and efficacy in teenagers remain under-researched. Thus far, most studies examining NRT results in this specific age group, manage smoking cessation by applying guidelines used for adult population, with slight reduction or modification in the therapy dosage. A Cochrane review that summarized the evidence for smoking cessation strategies in youth concluded that there were insufficient data to recommend a specific type of pharmaceutical treatment in young smokers (13). Nevertheless, there are guidelines that encourage the use of NRT in teenage smokers that are addicted to nicotine (regular smokers), but not in occasional smokers based on adult data (14, 15).

Policies governing the application of NRT in adolescents differ from those for adults as they often emphasize prevention of smoking initiation rather than cessation in adolescents (15). As such, resources and strategies for adolescent smoking cessation—including NRT—are less developed compared to those targeting adults, further widening the policy divide. As mentioned before, the long-term effects of nicotine on the developing brain are a significant concern. Research suggests that nicotine exposure during adolescence can negatively impact brain regions involved in decision-making, memory, and impulse control, raising ethical and safety concerns about encouraging NRT use in this population (8). Studies have shown mixed results, with limited success in achieving sustained smoking cessation among youth. This lack of data makes it difficult to formulate clear policies or guidelines for adolescents.

Social perceptions surrounding NRT further complicate its use among adolescents. Misconceptions about the safety and efficacy of NRT are prevalent among young people, including concerns about side effects, such as skin irritation from nicotine patches and the taste of nicotine gum, often described as “peppery,” which may stem from improper use (16). These negative perceptions contribute to reduced interest and adherence to NRT among young users. Moreover, youth often view NRT as less effective or unnecessary, preferring to attempt cessation without pharmacological assistance (“cold turkey”) or turning to alternative methods, such as electronic nicotine delivery systems, despite scepticism about their safety and efficacy (16, 17). Additionally, harm perceptions of nicotine itself play a significant role in tobacco use behaviour among youth. Evidence suggests that adolescents with greater harm perceptions of nicotine in cigarettes, e-cigarettes, and NRT are less likely to report current tobacco use, underscoring the influence of these perceptions on cessation attempts (18). However, among youth already using cigarettes or e-cigarettes, such harm perceptions do not predict transitions between these products, highlighting a complex interplay of factors influencing smoking behaviours in this age group. This implies that perceptions of nicotine harm may act as both protective and neutral factors depending on the stage of smoking behaviour. The stigma surrounding smoking cessation tools like NRT (e.g., being seen as a sign of weakness or dependence) may deter adolescents from using these products. Public perceptions and lack of awareness about the benefits of NRT further complicate its acceptance and use in this age group.

Therefore, it is important that clinical guidance and public health policy be grounded in robust evidence which confirms the efficacy and safety of NRT in younger populations, particularly those under 18. Most studies and guidelines rely on adapted adult protocols, with little attention given to age-specific factors such as developmental differences, social perceptions, and varying adherence rates. This review aims to address these gaps by providing a focused analysis of NRT’s role in adolescent smoking cessation and discussing potential future strategies for improving outcomes in this population. Additionally, the study highlights the rising prevalence of novel nicotine products, such as e-cigarettes, among adolescents and underscores the urgent need for research on new cessation tools tailored to this demographic. As these products continue to evolve and gain popularity, targeted cessation interventions that address both traditional tobacco and novel nicotine products are essential for reducing youth nicotine dependence.

## Methods

### Study design

The authors conducted a systematic review that adheres to the Preferred Reporting Items for Systematic Reviews and Meta-Analyses (PRISMA) guidelines (19).

### Eligibility criteria

This review includes randomized controlled trials (RCTs) and observational studies that have examined the efficacy and safety of NRT in adolescents aged 12-21 years. Smoking cessation or abstinence rates were considered as the primary endpoint, verified by self-report and biochemical validation. Secondary outcomes entailed withdrawal symptoms relief, reduction in cigarette consumption and adverse events. The following inclusion and exclusion criteria were applied:

*Inclusion criteria*

- Ages between 11-21 years old
- Smoking at least 1 cigarette per day
- Use of nicotine replacement products, either inhaler, chewing gum, patch, lozenge
- Full text availability
- English language

*Exclusion criteria:*

- Adults
- Pregnant adolescents
- No full text availability
- Case reports, case series, editorials, Cochrane reviews

### Information sources and search strategy

A detailed search of PubMed and Cochrane Library databases was conducted to find relevant studies published between January 1990 and August 2024. The search terms included combinations of key terms “nicotine replacement therapy”, “adolescents”, and “smoking cessation”, using the Boolean operators OR and AND where necessary.

### Data collection and analysis

Two independent reviewers (Ioannis Beis and Anastasios Dimou) initially screened titles and abstracts for relevance. Full texts of potentially eligible studies were retrieved and assessed for inclusion criteria. Discrepancies were resolved through discussion or consultation with a third reviewer (Athanasia Pataka).

### Data extraction and quality assessment

**Figure 1:**
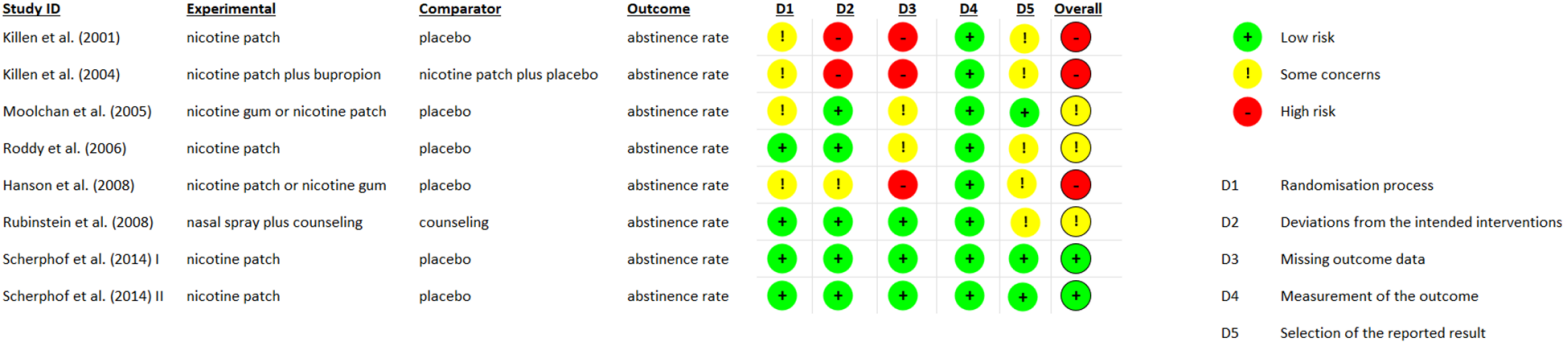
Quality assessment of RCTS with RoB 2 tool.

Data will be extracted using a standardized form including author, year of publication, study design, NRT specifics, duration of follow-up, outcome measures, and results. Quality assessment was performed using the Cochrane Risk of Bias Tool (RoB 2) for RCTs and the Newcastle-Ottawa Scale for observational studies (20) (21),. The updated version of RoB 2 provides a cohesive assessment of evidence from RCTs through a refined algorithm that answers specific signaling questions. The Newcastle-Ottawa Scale is an established method, with validity and inter-rater reliability, to evaluate quality of observational studies, and so an adapted form was used in our review for non-randomized and cross-sectional studies. The quality assessment process is depicted in **Figure 1** and **Table 1**.

**Table 1:** Newcastle-Ottawa Scale adapted for non-randomized studies and for cross-sectional studies.

*Studies*	Selection	Comparability	Outcome
*Non-randomized studies*			
Smith et al. (19)	***	*	**
Hurt et al. (20)	****		**
*Cross-sectional studies*			
Klesges et al. (22)	****		*
Haysom et al. (30)	*		*

Good quality: 3 or 4 stars in selection domain AND 1 or 2 stars in comparability domain AND 2 or 3 stars in outcome/exposure domain, Fair quality: 2 stars in selection domain AND 1 or 2 stars in comparability domain AND 2 or 3 stars in outcome/exposure domain, Poor quality: 0 or 1 star in selection domain OR 0 stars in comparability domain OR 0 or 1 stars in outcome/exposure domain.

## Results

### Study selection

**Figure 2:**
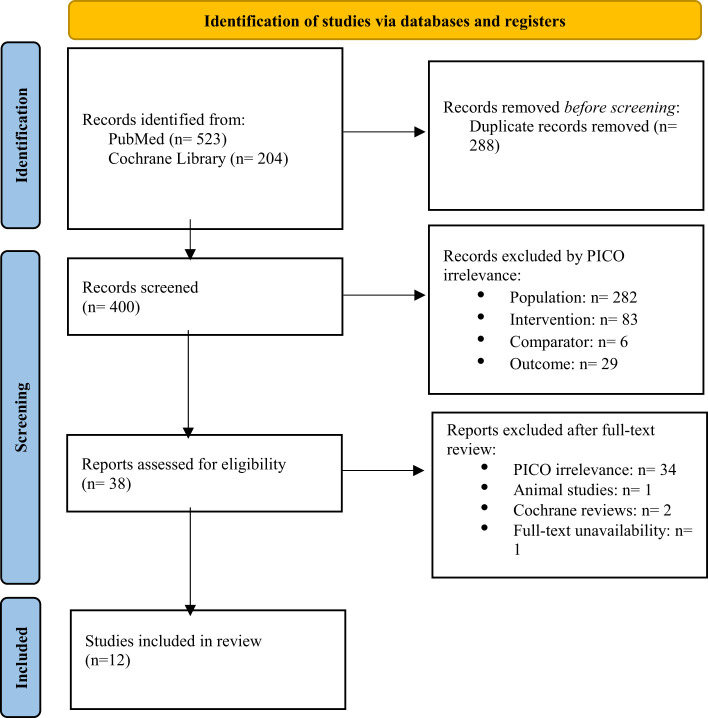
Flowchart of selection process.

The initial search yielded 727 records. After duplicates were removed, 400 records were screened, and 38 full-text articles were assessed for eligibility. Ultimately, 12 studies met the inclusion criteria, with 8 of those being randomized controlled trials (RCTs) (22–25, 27–29), 2 being non-randomized trials (30, 31), and 2 of them being cross-sectional studies (32, 33). The flow chart for the selection process is depicted in **Figure 2**.

### Study characteristics

The studies varied significantly not only in design, but also in population, geography and setting. Total participant number was 5,122 adolescents, while the study by Klesges et al. (25) consisted of 4,078 subjects. The age of participants ranged from 11 to 21 years, with an overall balanced gender distribution. Included studies were conducted across 4 countries, predominantly in the United States, United Kingdom, The Netherlands and Australia. The setting usually involved students attending high school, while there were studies that included adolescents from a shelter, and incarcerated youth. The duration of NRT ranged from 6 weeks to 12 weeks, with nicotine patches being the most common form of therapy, followed by gums and nasal spray. Study characteristics are included in **Table 2**.

**Table 2:** Study characteristics.

Author	Type of study	Sample characteristics	Intervention	Primary Endpoint (Abstinence Rate)	Secondary Endpoints	Adverse Events
Smith et al. (22)	Non-randomized, open-label trial	n=22 age=13-17 years old smoking status=20cpd	Nicotine patch (22mg for 6 weeks and 11mg for 2 weeks) plus counseling	14% at week 8, 4.5% at 6 months (confirmed by self-report and expired CO≤8ppm)	Decrease in nicotine withdrawal during week 1	Skin reaction (erythema), headache, nausea, tiredness
Hurt et al. (23)	Non-randomized, open-label trial	n=101 age=13-17 years old smoking status=20 cpd	Nicotine patch (15mg for 6 weeks) plus brief counseling Nicotine patch (15mg for an 8-hour session)	10.9% at week 6 and 5% at 6 months (confirmed by self-report and expired CO8ppm)	Decrease in nicotine withdrawal at week 2 through 6 from baseline Mean cotinine levels lower than baseline at weeks 3 and 6	Upper respiratory tract infections, headache, nausea and/or vomiting, skin reaction, sleep disturbance
Killen et al. (24)	RCT	n=92 age=16.9 years old smoking status=18.9 cpd	Nicotine patch (15mg for an 8-hour session)	Not measured	No withdrawal symptom relief Decrease in heart rate in the placebo group	Itching, dizziness, headache
Klesges et al. (25)	Cross-sectional	n=4078 age=16.8 years old smoking status: ≥ 1 cpd=258 participants	Nicotine patch or nicotine gum	Not measured	40% of former smokers used NRT to quit 18% of NRT users reported as never smokers	Not reported
Killen et al. (26)	RCT	n=211 age=15-18 years old smoking status=15.6cpd	Nicotine patch plus bupropion vs nicotine patch plus placebo (NRT for 8 weeks and bupropion for 9 weeks)	23% vs 28% at week 10 and 8% vs 7 % at week 26 (confirmed by self-report and expired CO < 9 ppm)	Decrease in cigarette consumption and cravings	Skin rash, nausea, dizziness, headache, digestive problems
Moolchan et al. (27)	RCT	n=120 age= 13-17 years old smoking status= 19cpd	Nicotine patch vs nicotine gum vs placebo (plus counseling) for 12 weeks	20.6% for patch, 8.7% for gum, 5% for placebo at 3 months after study completion (confirmed by self-report and expired CO ≤ 6 ppm)	Reduction in cpd for all 3 groups over 80% at the end of the treatment phase	Pruritus, erythema, headache, fatigue, insomnia, nausea, anxiety, sore throat, shoulder or arm pain, dizziness
Roddy et al. (28)	RCT	n=98 age= 11–21 years old smoking status= 10cpd	Nicotine patch for 6 weeks (15 mg/10 mg/5 mg for 2 weeks each) vs placebo	10% for patch group, 8% for placebo group at week 4	Smoking prevalence was 44%	Itching, rash pain or paresthesia at patch site dizziness
Hanson et al. (29)	RCT	n=103 age= 13–19 years old smoking status= 11.8cpd	Nicotine patch vs nicotine gum vs placebo for 4 weeks	6.8% and 4.9% at the 3-month and 6-month follow-up visits respectively (30-day period of abstinence)	At least 50% smoking reduction in 49.4% of participants Decrease in cotinine levels at the 3-month follow-up visit	Not reported
Rubinstein et al. (30)	RCT	n=40 age= 15–18 years old smoking status= 9.9cpd	Nicotine nasal spray plus counseling vs counseling alone for 8 weeks	No abstinence in the nasal spray group vs 11.8% in the only counseling group	No difference in withdrawal scale between the two groups No difference in craving	Nasal irritation and burning complaints about the taste and smell
Scherphof et al. (31)	RCT	n=257 age= 16.7 years old (mean) smoking status= 11-20 cpd (50% of participants)	Nicotine patch vs placebo for 6 or 9 weeks	31.9% after 2 weeks and 14.8% at the end of treatment	No end-of-treatment effect No difference in compliance rates between groups	Tiredness, cough, insomnia, itchiness and headache
Scherphof et al. (32)	RCT	n=257 age= 16.7 years old (mean) smoking status= 11-20 cpd (50% of participants)	Nicotine patch vs placebo for 6 or 9 weeks with extended follow up period (6 and 12 months)	8.1% of participants were abstinent at 6 months and 4.4% at 12 months	Not reported	Not reported
Haysom et al. (33)	Cross-sectional	n=252 age= 11–21 years old smoking status= 11-20cpd (36% of participants)	Nicotine patch for 2 weeks	Not reported	82.1% of participants smoked cigarettes prior to their current incarceration 38% of the smokers were nicotine dependent	Sleep issues

cpd; cigarettes per day, RCT; Randomized Controlled Trial.

### Efficacy of NRT

#### Cessation rates

The majority of studies showed no significant rates of tobacco abstinence at the end of treatment. Smith et al. (22) show that 19 out of 22 participants completed treatment, 12% achieved cessation at week 8, confirmed by expired air carbon monoxide levels, while only 1 participant was smoke free at 3 and 6 months. Similarly, a 10% of smoking abstinence rate at 6 weeks was presented by Hurt et al. and 5% at 6 months (23). Killen et al. (24) conducted a clinical trial comparing nicotine patch plus bupropion vs nicotine patch plus placebo which demonstrated abstinence rates of 23% and 28%, respectively, at week 10, and 8% and 7% at week 26 of treatment. A more recent study by Scherphof et al. (31), reported a significant effect of NRT in cessation rates 2 weeks after initiation, although it gradually wore off after treatment completion. Compliance rates varied notably among participants, while certain studies did not investigate cessation rates (24, 25, 33).

#### Reduction in smoking frequency

Authors record varying rates of decrease in cigarette consumption across studies. Killen et al. (26) used a random regression model which showed significant reduction in smoking frequency, with a prolonged post-treatment effect. Moolchan et al. (27) reported over 80% of decrease in smoking, although with no difference between the 3 groups that used nicotine patch, nicotine gum, or placebo. A clinical trial by Rubinstein et al. (30) using nicotine nasal spray reported no difference in cigarettes smoked per day in participants. Finally, Hanson et al. (29) showed that half of the adolescents achieved 50% reduction in baseline smoking, though it was not followed by a concomitant decrease in nicotine metabolite levels, such as cotinine and NNAL.

#### Withdrawal symptoms relief

Most studies evaluated withdrawal symptoms by either calculating nicotine withdrawal score after self-report of subjective symptoms, while one study used the Minesota Withdrawal Scale (30). Smith et al. found a significant decrease in weekly average withdrawal score from week 2 to week 8. Similarly, after an initial increase in nicotine withdrawal score at week 1, Hurt et al. (23) observed significant reduction from baseline at week 2 throughout week 6. In the study conducted by Killen et al. (24), two sessions of 8-hour nicotine patch vs placebo were applied to participants, but no statistical significance in withdrawal score between the two groups was reported. Last but not least, Rubinstein et al. (30), showed no difference in withdrawal symptoms and craving at week 8 between nicotine nasal spray plus counseling group and the counseling-only group.

#### Safety Profile of NRT

The most reported side effects associated with NRT were generally mild and included skin irritation at the patch site, nausea or vomiting, and headaches. Serious adverse effects were infrequent, with no reported cases of nicotine poisoning or cardiovascular events. For example, Smith et al. (22) observed that 68% of participants experienced skin reactions, with 55% reporting erythema. Other common side effects in this study included headaches (41%), nausea or vomiting (41%), tiredness (41%), dizziness, and arm pain (23%). Hurt et al. (23) similarly reported high incidences of upper respiratory infections (44%) and headaches (43%), although the frequency of nausea or vomiting was lower (13%). Skin reactions were noted in 12% of participants, while 10% experienced sleep disturbances. Killen et al. (24) found that 18% of participants reported itching, with lower frequencies of dizziness (1.3%) and headaches (0.02%). In a later study by Killen et al. (26), participants reported skin rash, nausea, dizziness, headaches, and digestive problems. Moolchan’s study (27) highlighted pruritus (17%), erythema (15%), and headaches (11.5%) as the most common side effects, along with fatigue (8%), insomnia (5%), nausea (4%), anxiety (3%), sore throat (3%), shoulder or arm pain (2%), and dizziness (2%) (24). Roddy’s research (28) indicated that itching was reported by 32% of participants compared to 14% in the control group, with rash (12% vs. 6%) and pain or paraesthesia at the patch site (12% vs. 8%) also noted. Dizziness was slightly more common in the control group (6% vs. 4%). Rubinstein (30) found nasal irritation and burning in 34.8% of participants, along with complaints about the taste and smell of the NRT (13%). Finally, Scherphof (31) reported tiredness, cough, insomnia, itchiness, and headaches as prevalent side effects.

## Discussion

The findings of this systematic review highlight the complex and nuanced efficacy of NRT as a smoking cessation tool among adolescents. Although nicotine replacement has been shown to be safe for short-term use in adolescents, it is not currently recommended as a component of pediatric tobacco use interventions (34). The American Academy of Pediatrics (AAP) highlights that NRT is safer than continued tobacco use, with contraindications primarily limited to hypersensitivity to nicotine or specific components of the medication, such as soy (35). Disease-related cautions, such as cardiovascular conditions or diabetes, are considered relative rather than absolute, emphasizing the importance of clinician judgment in weighing risks and benefits. Similarly, the American Academy of Family Physicians (AAFP) supports the safety of short-term NRT in adolescents, citing no biological evidence of significant harm and reinforcing that the benefits of quitting outweigh the risks of continued nicotine exposure (36). These positions align with the understanding that addressing the high disease burden of adolescent smoking or vaping, including concerns over pulmonary toxicity from e-cigarette chemicals, necessitates a pragmatic approach to smoking cessation interventions. Ultimately, while NRT is considered a safer alternative to continued tobacco use, its long-term safety profile in adolescents requires further investigation, and clinical discretion remains crucial in its application.

The overall cessation rates reported across the included studies suggest that NRT may have limited success in achieving long-term abstinence among adolescent smokers. For instance, Smith et al. and Hurt et al. both reported modest cessation rates, with only a small percentage of participants remaining smoke-free after treatment. Similarly, the study by Killen et al. (26) demonstrated initial improvements in cessation rates during the active treatment phase, but these rates significantly declined by the 26th week. This trend indicates that while NRT can temporarily reduce smoking, its ability to sustain long-term abstinence is questionable in the adolescent population. In contrast, adult studies highlight that NRT’s effectiveness improves with longer treatment durations and higher doses (37). While most adolescent trials have used NRT for less than 12 weeks and at lower doses than those recommended for adults, guidelines for the latter group recommend at least three months of treatment, with extensions as needed to prevent relapse (38). Extended use is supported by evidence showing that longer NRT durations result in higher cessation rates, while NRT remains safer than smoking (39–41). This, however, cannot be applied in adolescents due to the combination of limited data and the concerns about long-term effects of nicotine exposure. Addressing these gaps in research is crucial for optimizing NRT’s potential in this population.

The inconsistent adherence and compliance rates across the studies further complicate the interpretation of NRT efficacy. Some studies did not adequately assess compliance, and those that did, such as Moolchan et al. noted significant variations in participant adherence. This variability may contribute to the mixed outcomes observed and underscores the importance of consistent usage in achieving effective smoking cessation.

Despite the modest cessation rates, several studies reported a significant reduction in smoking frequency among adolescents using NRT. For example, Killen et al. (26) found a significant decrease in the number of cigarettes smoked per day, with effects persisting post-treatment. This suggests that NRT may be more effective in reducing smoking intensity rather than in achieving complete abstinence. However, the reduction in smoking frequency did not always correspond to a reduction in nicotine metabolite levels, as noted by Hanson et al. raising questions about the actual impact of NRT on nicotine dependence.

The relief of withdrawal symptoms is a critical component of NRT’s mechanism of action, yet the results in this area were also mixed. While studies like those by Smith et al. and Hurt et al. reported significant reductions in withdrawal symptoms over the course of treatment, others, such as Rubinstein et al. found no significant difference between the NRT and placebo groups. The variability in withdrawal symptom relief may be influenced by factors such as the type of NRT used, the dosage, and individual differences in nicotine dependence.

The safety profile of NRT in adolescents appears to be generally favourable, with most side effects being mild and manageable. Skin reactions, headaches, and nausea were the most reported adverse effects, consistent with findings in adult populations. Serious adverse effects were rare, and no instances of nicotine poisoning or cardiovascular events were reported across the studies, suggesting that NRT is a relatively safe intervention for adolescent smokers. However, the high incidence of skin reactions, particularly with nicotine patches, may affect adherence to therapy, as noted by Smith et al. Additionally, the occurrence of side effects such as dizziness and nausea could deter continued use, especially in a population that may already be ambivalent about quitting smoking. Therefore, while NRT is safe, its tolerability among adolescents requires careful consideration, and alternative forms or doses of NRT may need to be explored.

Currently, electronic cigarettes and novel tobacco products have infiltrated adolescents’ lives largely through the influence of social media and targeted advertising (3). These platforms often feature enticing and glamorized depictions of vaping, making it seem trendy and socially acceptable, particularly to impressionable teenagers (42). Some companies even resort to illicit methods, bypassing regulations by promoting their products through influencers and user-generated content, which is difficult to regulate (43). This tactic not only downplays the risks of addiction but also entices a younger demographic into a cycle of nicotine dependency under the guise of harm reduction, effectively grooming the next generation of lifelong consumers. One in 7 high school students has used tobacco in the past 30 days, according to the 2022 National Youth Tobacco Survey, with e-cigarettes being the most frequent form (44) and early initiation may predict subsequent smoking of conventional cigarettes (45). New nicotine products continue to evolve with higher nicotine concentrations and several flavours becoming available. Electronic cigarettes by vaping nicotine are not an acceptable option for smoking cessation and for the treatment of nicotine dependence. Although adolescents may use electronic cigarettes to vape nicotine in order to eliminate nicotine withdrawal symptoms and quit conventional cigarettes, this may promote nicotine dependence and other potential harms (46).

Apart from nicotine replacement therapy devices, electronic cigarettes have occasionally been used as smoking cessation tools, though they differ significantly in composition, regulation, and potential health risks. NRT devices are regulated medical products delivering controlled doses of nicotine to alleviate withdrawal symptoms without exposure to toxic compounds (47). In contrast, e-cigarettes heat a liquid containing propylene glycol, vegetable glycerin, and nicotine into an aerosol for inhalation.

Recent evidence has raised concerns about the toxicological risks associated with e-cigarette use. Studies have found that e-cigarettes can release several harmful compounds, including volatile organic compounds, heavy metals (such as arsenic, cadmium, nickel, and lead), and carcinogenic substances. These toxic agents, particularly metals, can originate from the heating elements, solder joints, and tobacco sticks in heated-tobacco-products (48). Emerging research also indicates that exposure to aerosols can induce mitochondrial stress, DNA damage, and deregulation of molecular pathways associated with cancer progression, respiratory diseases, and cardiovascular risks (49).

Regulatory approaches to e-cigarettes vary across countries. For example, in the UK, they are actively promoted as smoking cessation aids, whereas other European countries enforce stricter regulations, including bans on flavors and advertising (50). Overall, while both e-cigarettes and NRT products can support smoking cessation, the potential health risks of the former, especially among youth, require careful consideration.

While NRT could be a potential method for helping adolescents cease novel nicotine product use, there is currently limited evidence to support its effectiveness (51). No RCTs have definitively shown NRT’s efficacy in this context, highlighting a significant gap in research (52). Becker et al. (53) suggest NRT for adolescents with moderate to severe nicotine use disorder, citing its effectiveness in adult smoking cessation and the absence of significant harm in teens. The former authors advise using a combination of nicotine patch with a short-acting NRT to manage cravings. They also emphasize the importance of tailoring the initial dosage to the patient’s nicotine dependence, adjusting it to ease withdrawal symptoms, and conducting regular follow-ups to monitor cravings, NRT tolerance, motivation, and any mental health concerns. Two ongoing RCTs are recruiting participants to evaluate the effectiveness of behavioural interventions, in combination with NRT for vaping cessation among the youth population (54, 55).

This is reflected also in an important nuance between the United States Preventive Services Task Force (USPSTF) and AAP guidelines on NRT use in young population. The USPSTF states that there is insufficient evidence to evaluate the balance of harms and benefits on the interventions for smoking cessation among school-aged children and adolescents (56). On the other hand, the AAP recommends the use of NRTs off label in young smokers who are moderately or severely addicted to nicotine and motivated to quit states given to the severe harms of tobacco dependence and the effectiveness of NRT for adults (35). However, both admit to the importance for ongoing research, prevention strategies, neither recommends NRT outright, and both suggest that it is up to the clinician to offer the best possible cessation strategy for the patient. Adolescents experience many barriers to care, and we agree with encouraging research and promoting clinician efforts to offer adolescents the best possible cessation strategies for them.

Potential alternative treatments to NRT for adolescent smoking cessation include behavioral support and digital interventions. Programs such as motivational interviewing, cognitive-behavioral therapy, and group counseling emphasize the immediate negative health effects of smoking and teach coping strategies, showing modest improvements in quit rates, especially when delivered over five or more sessions (57). Social concerns and peer support also play a crucial role, as adolescents are more likely to quit when their peer group does not smoke (58). Digital tools, including text message-based interventions and mobile have gained popularity (59). For instance, interactive text messaging programs providing behavioral support have reported higher abstinence rates compared to control groups, though dropout rates remain high (60). Self-help resources, including websites and telephone counseling services provide additional support and education tailored to adolescents (61).

## Limitations

This systematic review has several limitations that should be considered when interpreting the findings. First, the variability in study design, population demographics, and NRT delivery methods across the included studies may limit the generalizability of the results. Second, most studies relied on self-reported smoking cessation outcomes, which could be subject to reporting bias, despite attempts at biochemical validation. Additionally, adherence to NRT was inconsistently reported, making it difficult to assess the true efficacy of the therapy. Many of the existing trials are relatively outdated and often focus on short-term interventions, using lower doses of NRT than those recommended for adults. Furthermore, few studies have assessed the long-term safety and effectiveness of NRT in adolescents or explored its use in combination with behavioral interventions. Future research should prioritize large-scale randomized controlled trials with extended follow-up periods to better understand the sustained impact of NRT in this population. It would also be valuable to investigate the role of newer cessation tools, such as digital interventions, and their integration with pharmacological treatments in adolescent smoking cessation strategies. The review also faced potential publication bias, as studies with negative or inconclusive results may be underrepresented in the literature. The relatively small sample sizes and short follow-up durations in many studies limit the ability to draw robust conclusions about the long-term effectiveness of NRT in adolescents. An important limitation of this review is the inclusion of studies with adolescent participants who may have underlying mental or physical health conditions or live in areas with adverse socioeconomic circumstances (28) (33),. These characteristics could influence the effectiveness of NRT and potentially bias the reported outcomes. For instance, adolescents with comorbid mental health disorders may experience greater challenges in achieving smoking cessation due to factors such as higher nicotine dependence or concurrent treatment for their condition. Similarly, physical illnesses, particularly those linked to smoking, may alter treatment efficacy. While excluding such studies might offer a more homogeneous sample, their inclusion provides a more comprehensive representation of the adolescent population.

## Conclusion

In conclusion, this systematic review underscores the challenges and limitations of NRT in promoting smoking cessation among adolescents. While NRT can reduce smoking frequency and alleviate withdrawal symptoms to some extent, its effectiveness in achieving long-term cessation is limited, and adherence remains a significant hurdle. The safety profile of NRT is acceptable, though the side effects may impact compliance. These findings suggest that while NRT can be part of the strategy for adolescent smoking cessation, it should be complemented by robust behavioural support and possibly tailored interventions that address the unique needs and challenges of adolescent smokers. Further research is needed to explore optimal dosing, delivery methods, and combination therapies that may enhance the effectiveness of NRT in this population.

## Data Availability

The original contributions presented in the study are included in the article/supplementary material. Further inquiries can be directed to the corresponding author/s.
